# Multimodal Retinal Imaging Classification for Parkinson's Disease Using a Convolutional Neural Network

**DOI:** 10.1167/tvst.13.8.23

**Published:** 2024-08-13

**Authors:** Alexander Richardson, Anita Kundu, Ricardo Henao, Terry Lee, Burton L. Scott, Dilraj S. Grewal, Sharon Fekrat

**Affiliations:** 1 Duke Eye Center, Department of Ophthalmology, Duke University School of Medicine, Durham, NC, USA; 2 iMIND Research Group, Duke University School of Medicine, Durham, NC, USA; 3 Department of Computer Science, Duke University, Durham, NC, USA; 4 Department of Neurology, Duke University School of Medicine, Durham, NC, USA

**Keywords:** machine learning, Parkinson's disease, optical coherence tomography angiography, fundus photography, ganglion cell–inner plexiform layer thickness color maps

## Abstract

**Purpose:**

Changes in retinal structure and microvasculature are connected to parallel changes in the brain. Two recent studies described machine learning algorithms trained on retinal images and quantitative data that identified Alzheimer's dementia and mild cognitive impairment with high accuracy. Prior studies also demonstrated retinal differences in individuals with PD. Herein, we developed a convolutional neural network (CNN) to classify multimodal retinal imaging from either a Parkinson's disease (PD) or control group.

**Methods:**

We trained a CNN to receive retinal image inputs of optical coherence tomography (OCT) ganglion cell–inner plexiform layer (GC-IPL) thickness color maps, OCT angiography 6 × 6-mm en face macular images of the superficial capillary plexus, and ultra-widefield (UWF) fundus color and autofluorescence photographs to classify the retinal imaging as PD or control. The model consists of a shared pretrained VGG19 feature extractor and image-specific feature transformations which converge to a single output. Model results were assessed using receiver operating characteristic (ROC) curves and bootstrapped 95% confidence intervals for area under the ROC curve (AUC) values.

**Results:**

In total, 371 eyes of 249 control subjects and 75 eyes of 52 PD subjects were used for training, validation, and testing. Our best CNN variant achieved an AUC of 0.918. UWF color photographs were the most effective imaging input, and GC-IPL thickness maps were the least contributory.

**Conclusions:**

Using retinal images, our pilot CNN was able to identify individuals with PD and serves as a proof of concept to spur the collection of larger imaging datasets needed for clinical-grade algorithms.

**Translational Relevance:**

Developing machine learning models for automated detection of Parkinson's disease from retinal imaging could lead to earlier and more widespread diagnoses.

## Introduction

Parkinson's disease (PD) is a neurodegenerative disorder exhibiting motor and non-motor features affecting 3% of adults over age 65.^1^ Loss of dopaminergic cells in the substantia nigra, associated with cardinal motor symptoms including bradykinesia, rigidity, and a resting tremor, is the hallmark of PD. When cardinal motor symptoms develop, over 80% of nigrostriatal dopaminergic cells are already affected.^2^

Multiple medications, such as levodopa and carbidopa, lessen PD symptoms, yet there is no pharmacologic cure. Medications for PD symptoms are more effective when administered earlier in the disease course; thus, earlier diagnosis and treatment may delay symptom progression and improve quality of life.^3^ The standard of PD diagnosis remains the postmortem histological presence of α-synuclein–positive inclusions in neuronal cells.^4^ Various diagnostic genetic, molecular, and biochemical biomarkers of PD have been studied; however, no definitive biomarkers have been identified thus far that provide a high-fidelity model of PD disease identification or any prediction of course.^5^

A clinical diagnosis of PD is currently made by clinical assessment in conjunction with scores and tools that aim to standardize evaluation, such as the Unified Parkinson's Disease Rating Scale.^6^ Even with standardized scales, an effect of interprovider variability in assessment and evaluation is observed that varies across the PD continuum. Studies have shown that PD diagnosis in the earlier stages may have a diagnostic accuracy as low as 26%.^7^ Given the devastating effects of PD on quality of life, as well as the lack of a cure, it is not surprising that a large unmet need exists for specific, cost-effective, and non-invasive tools to assist in PD screening, diagnosis, and monitoring.

Changes in retinal structure and vasculature have been associated with neurodegenerative changes in the brain.^8^^,^^9^ Cross-sectional studies in PD using optical coherence tomography (OCT) and OCT angiography (OCTA) imaging have revealed a decrease in retinal perfusion density, vessel density, and choroidal vascularity index in patients with PD compared to cognitively normal controls without PD.^9^ Another study revealed increased retinal venule tortuosity in PD patients compared to controls using ultra-widefield (UWF) scanning laser ophthalmoscopy (SLO).^10^ An investigation into OCT features found that ganglion cell–inner plexiform layer (GC-IPL) and inner nuclear layer thinning was significantly associated with a PD diagnosis.^11^ Further, longitudinal retinal imaging data have suggested that there may be significantly faster neuronal and microvascular loss over time in individuals with PD, suggesting its prognostic potential.^12^

The use of machine learning in PD diagnosis is a rapidly growing area of interest. Retinal imaging is a commercially available and broadly accessible imaging type with significant translational potential.^13^^–^^21^ Herein, we developed a convolutional neural network (CNN) model with the goal of identifying PD using solely multimodal retinal images comprised of OCT, OCTA, and UWF SLO images.

## Methods

### Participants

This study (clinicaltrials.gov identifier NCT03233646) was approved by the Duke Health Institutional Review Board (Pro00082598). The study followed the Health Insurance Portability and Accountability Act of 1996 and adhered to all tenets of the Declaration of Helsinki. Written informed consent was provided by all study subjects or their legally authorized representative prior to study participation.

Study participant images were collected from 2017 to 2023 from the existing Eye Multimodal Imaging in Neurodegenerative Disease (iMIND) database. Prior to enrollment, all patients with PD were evaluated by an experienced movement disorders specialist (BLS), and the clinical diagnosis of PD was confirmed according to the clinical criteria of the International Parkinson and Movement Disorder Society.^22^ The control group consisted of cognitively normal individuals from the iMIND database. Subjects with prior retinal surgery, high myopia, or hyperopia (greater than 6 diopters in magnitude), significant head tremor, diabetes, uncontrolled hypertension, glaucoma, history of retinal or optic nerve pathology, or corrected visual acuity less than 20/40 on the day of image acquisition were excluded. Study participants also underwent cognitive function evaluation by trained iMIND study personnel on the day of image acquisition using the Mini-Mental State Examination (MMSE), an 11-question measure that tests five areas of cognitive function.^23^

### Image Acquisition

Non-mydriatic images were acquired by experienced iMIND study personnel using the ZEISS CIRRUS 5000 Spectral-Domain OCT with AngioPlex software (version 11.0.0.29946; Carl Zeiss Meditec, Dublin, CA), and layer segmentation was done using the integrated ZEISS software. Active retinal motion tracking software was used to reduce motion artifact during acquisition. Images were each reviewed manually by trained study personnel (AK, DSG) for quality assessment. Images with lower than 7/10 signal strength index, motion artifact, segmentation artifact, shadow artifact, or focal signal loss were excluded. These designations were designed to exclude images where analysis would be hindered by the severe artifact. Only subjects that had good-quality images as determined by trained iMIND study staff (AK, SF, DSG) for all four image inputs, GC-IPL color thickness maps, 6 × 6-mm OCTA superficial capillary plexus images, UWF color photographs, and UWF autofluorescence (FAF) photographs, were included in the analysis.

### GC-IPL Thickness Color Maps

One 512 × 128-µm macular cube scan was acquired for each eye, and the GC-IPL thickness color map was exported in tagged image file format (TIFF) for analysis.

### OCTA Scans

For each eye, a 6 × 6-mm OCTA scan centered on the fovea was acquired, and the superficial capillary plexus (SCP) image was exported in bitmap format for analysis.

### UWF Fundus Color and FAF Photographs

UWF fundus color and FAF photographs were obtained by trained study personnel using nonmydriatic SLO (Optos California; Optos, Marlborough, MA). Fundus photos with peripheral distortion or motion and eyelid artifact within the major vascular arcades were excluded.

### Model Development

In total, 296 control eyes (80%) were used for training, 36 (10%) for validation, and 39 (10%) for testing; 59 PD eyes (79%) were used for training, six for validation (8%), and 10 (14%) for testing. Two hundred and four eyes of control subjects and 39 eyes of PD subjects were used for development and testing of the model that considered only right eyes, randomly assigned to an 80% train, 10% validation, and 10% test split. Also, 169 eyes of control subjects and 36 eyes of PD subjects were used for development and testing of the model that considered only left eyes, randomly assigned to an 80% train, 10% validation, and 10% test split.

Deep learning is a branch of machine learning that uses neural networks with multiple layers to model complex patterns. Three separate deep-learning models were developed, using images from the right eye only, left eye only, or both eyes. The single-eye models were developed because some subjects had only one imageable eye or a poor image quality for one eye. All models were trained to receive inputs of GC-IPL thickness color maps, OCTA SCP 6 × 6-mm en face images, and UWF fundus color and FAF photographs to produce a score suggesting whether a subject carried a clinical diagnosis of PD.

For dataset preprocessing, all images were resized with Python Imaging Library to 256 × 256 pixels and converted to 224 × 224 PyTorch tensors to be compatible with the VGG19 architecture. For dataset augmentation, all images were subjected to random horizontal and vertical flips with a probability of 0.5. For all models, an 80% train, a 10% validation, and a 10% test split were used. To ensure that there was no overlap among the training, validation, and test groups, random assignment was done by patient ID so that eyes of the same subject were assigned to the same group (i.e., training, validation, or test). The model parameters with the best performance on the validation split were selected for testing. The median area under the receiver operating characteristic (ROC) curve (AUC) of 10 model training runs was selected.

**Figure 1.**
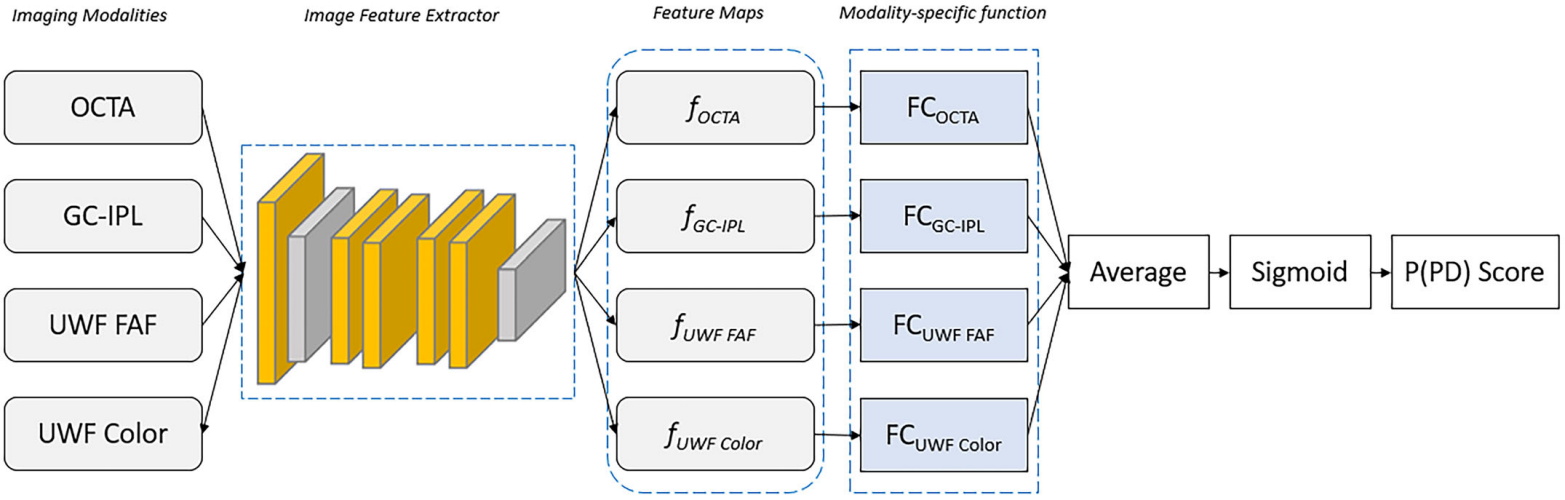
Illustration of the PD classification model architecture. The model leverages a pretrained VGG19 CNN as a shared-weight image feature extractor (shown in the *blue dotted box*) to generate feature maps for each imaging modality, then feeds the results to modality-specific functions. The model averages the outputs from these functions and converts the result into a PD probability score in the [0, 1] range to obtain a final prediction result.

The model consisted of a shared pretrained convolutional encoder, parallel image modality–specific feature transformations, and prediction heads for each modality that converge into a single output (Fig. 1). Transfer learning was leveraged from a VGG19 model pretrained on ImageNet as the convolutional encoder.^24^ Pretraining is a machine learning technique where a model is trained on a large general dataset so it can learn basic image feature extraction such as edge detection, and transfer learning is where the pretrained neural network is fine-tuned to a smaller and specific problem, such as Parkinson's disease classification with retinal imaging. The images were then processed through the convolutional encoder, before being fed to image modality–specific feature transformations consisting of three fully connected layers: a dropout layer, a batch normalization function, and a rectified linear unit (ReLU) activation function. The outputs of these parallel feature transformations were averaged and processed through a sigmoid activation function to produce a total model output. The model was trained for 50 epochs using the adaptive moment estimation (ADAM) optimizer^25^ with a weight decay of 0.01. Two methods were used to counteract the imbalanced training dataset, which contained fewer PD eyes than control eyes: a modified binary cross entropy loss function and a weighted random sampler. The weighted random sampler oversampled PD samples to create a 1:1 class ratio in training batches.

### Statistical Analysis

The average age of the cognitively normal control cohort was compared with that of the PD cohort using an independent sample *t*-test. As a performance metric, we considered the ROC curve, the AUC, sensitivity, and specificity. Bootstrapped ROC computations with 10,000 samples were used to estimate the 95% confidence intervals (CIs) for the AUC values. To convert the model outputs into a clinical classification (PD vs. control), we thresholded the model scores with the label set to optimize the Youden index,^26^ a classification measure specifically designed for medical contexts. The model results were compared for each imaging modality as the sole input to provide information on their relative efficacy and contrast with the full model. The DeLong test, a statistical method to compare the predictive power of different classification algorithms, was used to compare the performance metrics for different inputs.^27^ We compared the predictions for a model using each imaging modality as the sole input to the model utilizing all inputs for both the right eye and left eye models and averaged the DeLong test results. A higher DeLong test *P* value and a higher AUC for a single feature model can be interpreted as a proxy of significant predictive power, as the predictions of the single feature model are not statistically significantly different from the predictions of the model processing all imaging types.

## Results

A total of 371 eyes (204 right and 169 left) of 249 control subjects and 75 eyes (39 right and 36 left) of 52 PD subjects were included for development and testing of the model that considered both right and left eyes. The PD cohort had a mean age of 65.9 ± 9.5 years, and the control cohort had a mean age of 66.4 ± 9.1 years (*P* = 0.656). The PD cohort had 31 males and 21 females, and the control cohort had 67 males and 184 females (*P* < 0.05).

**Figure 2.**
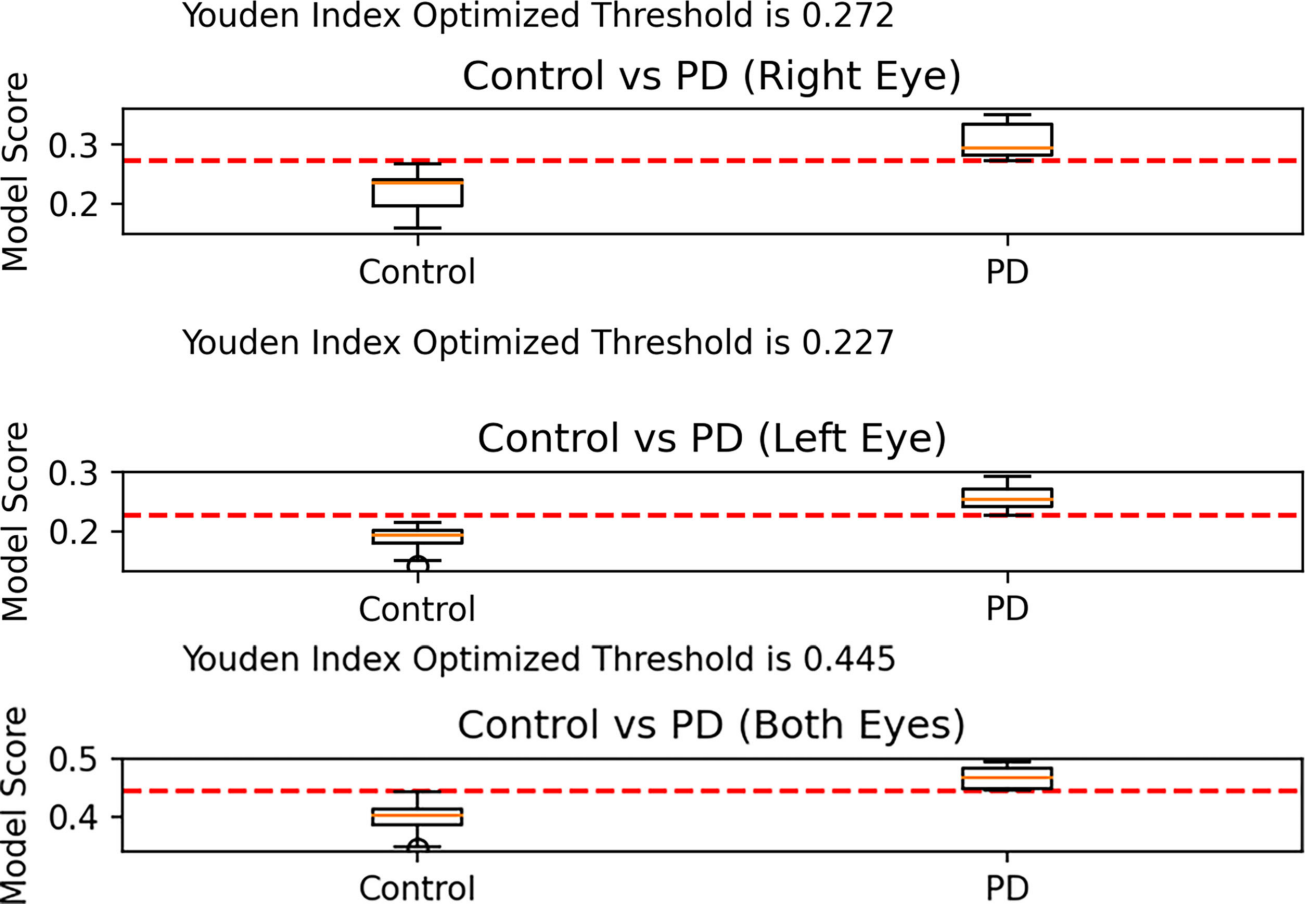
Model outputs for each eye in the PD and control test sets evaluated by the right-eye-only, left-eye-only, and both eyes models. The *red dashed line* indicates the decision boundary of the model for its output probabilities. Less overlap between the box plots for the control and PD groups indicates a model had better classification performance, and points on the box plot closer to the threshold (*red dashed line*) represent patients that the model classified with lower confidence.

**Figure 3.**
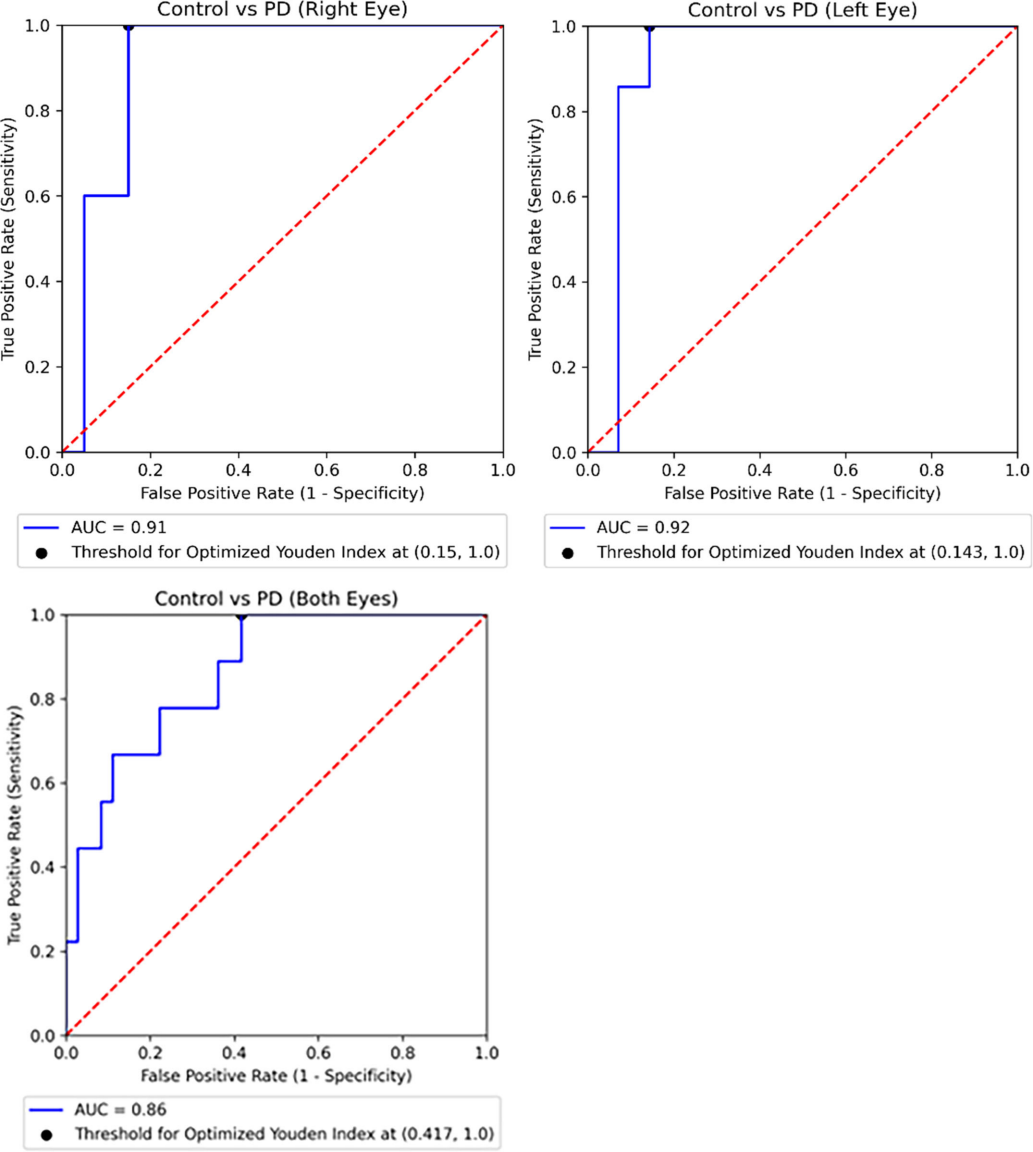
ROC curve for the right-eye-only, left-eye-only, and both eyes models using all features on the test set.

**Figure 4.**
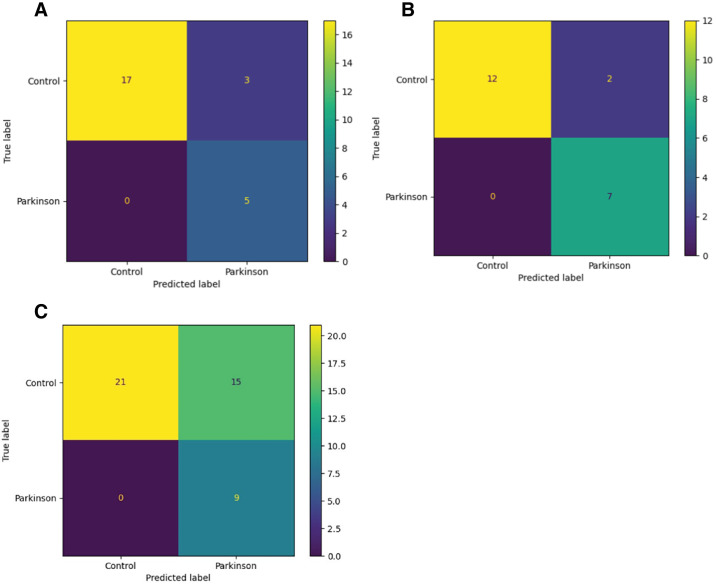
Confusion matrices for the right eye only (**A**), left eye only (**B**), and both right and left eyes (**C**).

**Figure 5.**
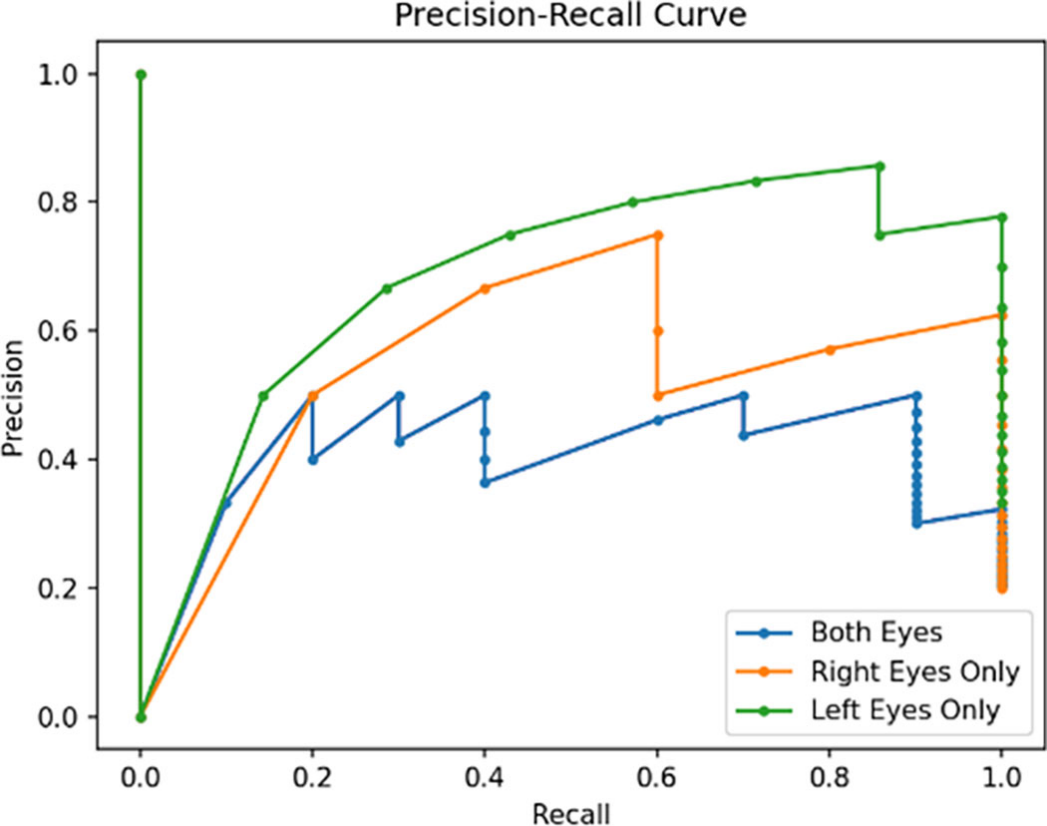
Precision–recall curves for each of the three models incorporating all image types. The AUC value is 0.405 for the model utilizing both eyes, 0.535 for the model utilizing right eyes, and 0.677 for the model utilizing left eyes only.

The Table provides the AUC values for the right, left, and both eyes models, as well as the AUC values for the model trained on each modality individually. Each model used the structure described in Figure 1, but with various input combinations as outlined in the Table. The best performing models used all inputs, including GC-IPL, OCTA, UWF color, and UWF FAF. The right-eye-only model had an AUC of 0.910 (95% CI, 0.798–1), and the left-eye-only model had an AUC of 0.918 (95% CI, 0.787–1) when including all inputs. The model using both eyes with all imaging modalities had an AUC of 0.861 (95% CI, 0.750–0.955). The right-eye-only model achieved a sensitivity of 100% and a specificity of 85%. The left-eye-only model achieved a sensitivity of 100% and specificity of 85.7%. The UWF color image was the best single input (right-eye-only AUC = 0.798, left-eye-only AUC = 0.838), followed by OCTA (right-eye-only AUC = 0.711, left-eye-only AUC = 0.811), and UWF FAF (right-eye-only AUC = 0.682, left-eye-only AUC = 0.794). GC-IPL thickness maps (right-eye-only AUC = 0.630, left-eye-only AUC = 0.556) were the worst performing single input. Figure 2 demonstrates model scores for each eye in the PD and control test sets evaluated by the three eye models including all inputs. Figure 3 displays the ROC curves for the right-eye, left-eye, and both eyes models with all inputs. The ROC curves in Figure 3 were also used to calculate the Youden index threshold values shown in Figure 2, which functioned as the decision point to determine sensitivity and specificity.^26^ Figures 4A and 4B show the confusion matrices, demonstrating how eyes in the test sets were classified by the models using the Youden thresholds. Figure 4C shows the confusion matrix for the model considering both right and left eyes. Figure 5 displays the precision–recall curves, which are often employed for binary classifiers on datasets with class imbalances, for each of the three eye models including all inputs.^27^ The AUC values for each of the precision–recall curves were 0.405, 0.535, and 0.677 for the models utilizing both eyes, right eyes only, and left eyes only, respectively.

**Table.** AUC on the Test Set Describes the Performance of Each Model in Predicting a Symptomatic PD Diagnosis Given the Imaging Inputs Described in the Left Column

Imaging Modalities Included	Right Eye Only, AUC (95% CI)	Left Eye Only, AUC (95% CI)	Right and Left Eyes, AUC (95% CI)	*P*, DeLong Test Result
OCTA, GC-IPL, UWF FAF, UWF color	0.910 (0.798–1.0)	0.918 (0.787–1.0)	0.861 (0.750–0.955)	N/A
OCTA	0.711 (0.471–0.929)	0.811 (0.529–1.0)	0.650 (0.436–0.842)	0.36
GC-IPL	0.630 (0.421–0.826)	0.556 (0.275–0.822)	0.625 (0.395–0.837)	0.05
UWF FAF	0.682 (0.500–0.857)	0.794 (0.588–0.959)	0.855 (0.674–1.0)	0.22
UWF color	0.798 (0.632–0.934)	0.838 (0.625–1.0)	0.805 (0.695–0.905)	0.43

To interpret the results, compare the AUC values to assess the effectiveness of each model; higher AUC values indicate better performance. The DeLong test results were derived by comparing the predictions of the right- and left-eye-only models using each imaging modality in isolation compared to using all imaging, then averaging the *P* values. A lower DeLong test *P* value indicates that the predictions of the model were more distinct compared to the model that used all inputs.

## Discussion

Our findings demonstrate that using deep learning applied to retinal imaging for identifying Parkinson's disease yields strong predictive performance. The right-eye, left-eye, and both eyes models used GC-IPL thickness color maps, en face OCTA images, and UWF color and FAF images to achieve AUCs of 0.910, 0.918, and 0.861, respectively, when applied to an independent test set. Because the 95% CI for the both eyes model closely overlapped with the 95% CIs for the right-eye and left-eye models, we cannot conclude that the single-eye models are superior, matching the findings of our previous work into the interocular symmetry of vasculature biomarkers in OCTA.^28^ Similarly, we cannot conclude that there are differences between the right-eye-only and left-eye-only models when each imaging input was isolated due to their closely overlapping CIs. The precision–recall curves for the three models that used all images aligned closely with each other and did not show evidence of extreme class imbalance in prediction accuracy. Our research group has previously published CNN models capable of using GC-IPL, OCTA, and UWF images, along with quantitative data, to predict Alzheimer's disease (AD) and mild cognitive impairment (MCI) diagnosis with high accuracy.^20^^,^^21^ The performance qualities were similar to the performance demonstrated in our previously developed CNN for classification of AD and control subjects using multimodal retinal images, but our PD model uses only images and does not include quantitative data.^21^

Previous studies have established a link between PD pathology and findings in the optic nerve and retina. Thinning of the peripapillary retinal nerve fiber layer (RNFL), foveal thinning, and outer plexiform layer thickening have been observed in the eyes of PD subjects.^29^^,^^30^ In Parkinson's disease, α-synuclein has been found in neuronal inclusions in the ganglion cell layer and inner nuclear layer.^31^^,^^32^ The loss of dopaminergic neurons in the substantia nigra has also been associated with RNFL thinning that may correlate with PD severity.^33^ It is possible that the CNN presented in this paper may be recognizing thinning in the RNFL or ganglion cell layer as a visual indicator of PD.^29^^,^^30^ Current evidence also indicates a chain of vascular events observed in postmortem tissue and preclinical models of PD that might manifest in the capillary network of the retina.^34^ The loss of dopaminergic neurons in PD widens the close contacts between neurons and retinal vasculature, impairing the blood–retinal barrier and triggering angiogenesis, but angiogenesis fails and vascular regression occurs.^34^^,^^35^ The retinal capillaries of patients with PD compared to controls are fewer in number, shorter in length, and larger in diameter.^35^

Machine learning–based diagnostic tools using retinal image inputs also have systemic disease utility. Retinal microvascular analysis has shown promise in identifying cardiovascular disease risk, including predicting risk factors such as blood pressure and coronary artery calcium score.^36^ Algorithms using retinal photographs to identify diabetic retinopathy can identify symptoms and predict progression in individual patients, serving as a non-invasive tool for diabetes management.^37^^–^^41^ Machine learning models that work with widely accessible data, such as retinal photographs obtained in primary care and optometry clinics, have impactful translational potential in medicine, as the pool of patients is significantly larger than in ophthalmology. The efficacy of UWF color images as a single input in our PD algorithm is therefore particularly important, given the existing widespread availability of UWF imaging devices and the potential of rapid deployment for point-of-care testing.

Our limited dataset size constrained the complexity of our model architecture. Complex neural networks with numerous parameters require larger training datasets to minimize overfitting, which may impair the ability to generalize to new samples. Models such as the CNN created herein would benefit from larger datasets to fully extract features, especially for complex or subtle changes such as retinal vasculature and nerve architecture differences in PD patients. Although we leveraged techniques such as transfer learning from a pretrained VGG19 neural network, weighted random sampling, and a weighted loss function, the performance of our model was still constrained by the dataset size. The manual grading of images required for diagnosis and assessment of image quality is a time-intensive component, but this may be mitigated by using algorithms such as the model developed by iMIND to automate and standardize image quality assessment.^42^ Another limitation was class imbalance, as there were fewer PD eyes than control eyes. A dataset with an imbalanced class distribution is a hindrance to classification, especially of the minority class, but our model consistently had better performance for PD eyes, with a sensitivity near 100% for most model variations. This can be a useful attribute, as sensitivity is preferred over specificity in the clinical screening setting.

The current PD model can classify patient retinal imaging as belonging to a PD group or a control group. We hope that our proof-of-concept model will encourage the development of the larger datasets needed to train clinical-quality algorithms and longitudinal datasets with subjects who have developed PD from a neurological normal state. Longitudinal datasets are particularly important, because they could be used to build deep-learning models that identify early-stage PD.

A similar model could be trained to predict the development of PD sequelae. Another future direction is combining previous classification algorithms for MCI and AD with this PD algorithm to screen for multiple neurodegenerative diseases.^20^^,^^21^ Continued recruitment and imaging of PD and control subjects, using standardized retinal imaging protocols, to build a larger training set will also improve the performance of the current CNN.

The model described herein has potential for clinical applications in assisting PD risk stratification. It also pushes the frontiers of this emerging field of oculomics, where retinal phenotyping and machine learning converge to allow scientists and clinicians to better understand the pathophysiology and diagnosis of neurodegenerative diseases including PD. Our pilot model herein serves as a proof of concept to spur the growth of larger datasets needed for advancements in predictive PD models and combined MCI, AD, and PD classifiers.
